# Prevalence and characteristics of pain in moderate-to-severe obstructive sleep apnea patients and effect of CPAP treatment

**DOI:** 10.1038/s41598-023-42967-5

**Published:** 2023-09-21

**Authors:** Chong Shen, Yanru Ou, Ruoyun Ouyang, Dandan Zong

**Affiliations:** Department of Respiratory and Critical Care Medicine, Second Xiangya Hospital, Central South University, Changsha, 410011 China

**Keywords:** Health care, Medical research

## Abstract

Pain problems are common in patients with obstructive sleep apnea (OSA), but few studies have thoroughly evaluated pain in these patients. The objective of this study was to examine the prevalence and characteristics of pain in moderate-to-severe OSA patients and the effect of continuous positive airway pressure (CPAP) treatment. Moderate-to-severe OSA patients and healthy controls (HC) completed the Short Form McGill Pain Questionnaire (SF-MPQ) and a portion of the Brief Pain Inventory (BPI) Short Form to assess pain characteristics. The Epworth Sleepiness Scale (ESS), the Short Form-36 (SF-36), and the Hospital Anxiety and Depression Scale (HADS) were used to measure daytime sleepiness, health-related quality of life (HRQoL), and psychological status, respectively. The OSA patients with pain were divided into a CPAP-treated group and a CPAP-untreated group based on their adherence to CPAP. The subjects' pain intensity was reassessed after 3 months. The prevalence of pain was 57.5% in OSA versus 27.1% in HC (*p* < 0.001). Head (39.0%) accounted for the highest proportion of overall pain locations in subjects with OSA, with 28.8% of OSA patients experiencing headaches. Pain in OSA was associated with impaired HRQoL and psychological problems. Patients with very severe OSA had an increased risk for pain problems (OR: 7.000,* p* = 0.041). Associated factors for pain intensity in OSA included age, ESS ≥ 9.0, and lowest pulse oximetry (LSpO2) < 80.0%. Pain intensity in OSA decreased significantly after CPAP treatment (*p* < 0.001). Pain was prevalent among patients with moderate-to-severe OSA and was associated with depression, anxiety, and a lower HRQoL. Patients with very severe OSA had an increased risk for pain problems. The intensity of pain in OSA can be predicted by age, ESS ≥ 9.0, and LSpO2 < 80.0%, and it can be alleviated through CPAP treatment.

## Introduction

Obstructive sleep apnea (OSA) is a prevalent condition characterized by complete or partial upper airway obstruction during sleep, leading to fragmented sleep patterns and recurrent nocturnal hypoxia. Individuals with OSA commonly experience psychological difficulties, increased daytime sleepiness and fatigue, impaired concentration and memory, as well as other symptoms^1^. Moreover, OSA has been associated with various health complications, such as diabetes, hypertension, and cardiovascular disorders^2^.

OSA and pain exhibit a strong association^3^. Numerous painful conditions such as headaches, temporomandibular disorders, and fibromyalgia are influenced by OSA and prone to development and exacerbation^4^. A recent study revealed that a significant number of patients with high-impact chronic pain, who were referred for specialized pain management also presented with OSA^5^. The influence of sleep deprivation and intermittent hypoxia has been combined to be the effects of OSA on pain, according to a theory^6^. Furthermore, OSA patients have also been reported to have greater odds of comorbid moderate and severe pain^7^. However, the prevalence and characteristics of pain in individuals with obstructive sleep apnea (OSA) remain inadequately understood, with limited studies comparing the experience of pain between OSA patients and healthy individuals. Furthermore, the association between significant clinical features of OSA, such as disrupted sleep structure, hypoxemia, microarousals, daytime drowsiness, and pain, has not been extensively investigated. Therefore, it is imperative to conduct a more detailed examination of the relationship between OSA and pain outcomes.

Due to the intricate nature of pain, involving both psychological and physical elements, it is often associated with heightened emotional distress and a reduced quality of life^8^. Research has found that individuals with OSA frequently encounter psychological symptoms such as despair and anxiety in addition to the typical excessive daytime sleepiness^9^. Moreover, the literature has already reported on the effects of OSA on health-related quality of life (HRQoL)^10^. A recent study has highlighted the pervasive nature of pain among primary care patients, significantly impacting their overall quality of life^8^. However, it remains unknown how pain affects the health-related quality of life (HRQoL) and psychological well-being of individuals with obstructive sleep apnea (OSA). To date, no studies have investigated this aspect.

Continuous positive airway pressure (CPAP) treatment is a well-established therapeutic approach for obstructive sleep apnea (OSA), yet limited research has been conducted on its impact on pain relief^11^. Therefore, our study aimed to investigate the prevalence, intensity, and location of pain in patients with moderate-to-severe OSA, explore the physiological functioning, psychological symptoms, and quality of life in OSA patients experiencing pain, examine significant clinical features associated with worse pain outcomes in OSA patients, and evaluate the effects of CPAP treatment on pain outcomes among individuals with moderate-to-severe OSA.

## Materials and methods

### Participants

Consecutive patients referred for suspected OSA at the Sleep Disorders Center of the Second Xiangya Hospital of Central South University were screened from September 2021 to September 2022. The OSA diagnosis was based on the International Classification of Sleep Disorders, third edition^11^. The exclusion criteria were: (1) AHI (apnoea hypopnea index) < 15 (events/hour); (2) diagnosis of complication except for hypertension, hyperlipemia, hyperuricaemia, and hypercapnia; (3) severe chronic debilitating conditions or pregnancy; (4) concurrent use of painkillers; and (5) inability to complete questionnaires.

Healthy, age-matched control participants were recruited from the Health Management Center of the Second Xiangya Hospital of Central South University. Except for possible obesity, all control subjects had a normal physical examination and laboratory tests. Control subjects were excluded from the study if they (1) were diagnosed with any kind of sleep-disordered breathing through polysomnography; (2) had concurrent use of painkillers; or (3) were unable to complete questionnaires.

### Polysomnography (PSG)

All participants received diagnostic PSG (Embla S4000; Medcare Technologies, Fuquay Varina, NC, USA). The monitoring was conducted at a specialized sleep center. Non-OSA, mild OSA, moderate OSA, and severe OSA were defined based on the Apnea–Hypopnea Index (AHI) as < 5, 5–15, 15–30, and ≥ 30 events per hour, respectively. Additionally, within the severe OSA category, a subgroup known as very severe OSA was defined with an AHI ≥ 60 events per hour^11^.

### Data collection of questionnaires

Data on baseline medical history, anthropometric, polysomnographic parameters and common questionnaires were collected. For all of the participants, demographic details (sex, age, BMI, diagnosis, smoking history, comorbidities, and medication history) were recorded. All participants completed the Epworth Sleepiness Scale (ESS) questionnaire, and excessive daytime sleepiness was defined as an ESS score ≥ 9^12^. The Short Form McGill Pain Questionnaire (SF-MPQ) was used to evaluate the pain intensity of subjects, which was divided into three parts: the Pain Rating Index (PRI), the Present Pain Intensity (PPI) index and the visual analogue scale (VAS). Based on pain intensity, the PPI is categorized into six levels: 0—no pain, 1—mild, 2—discomforting, 3—distressing, 4—horrible, and 5—unbearable. The three pain scores are derived from the sum of the intensity rank values of the words chosen for sensory, affective, and total descriptors, with a higher score indicating greater pain intensity^13,14^. The Brief Pain Inventory (BPI) was used by the subjects to locate the pain on the body chart^15^.

The subjects with OSA also completed the following questionnaires: The Hospital Anxiety and Depression Scale (HADS) was used to measure the severity of anxiety and depression, with a higher score indicating greater anxiety or depression^16^. The Short Form-36 (SF-36) was used to evaluate HRQoL. The scores of the SF-36 test are separated into 8 categories (physical functioning, role physical, bodily pain, general health, vitality, social functioning, role emotional, mental health, and health transition), with lower scores indicating worse HRQoL^17^.

### Follow-up of OSA patients with pain

The OSA patients with pain were divided into a CPAP-treated group and a CPAP-untreated group based on their adherence to CPAP. Patients undergoing CPAP therapy were telephonically followed up on a weekly basis. The follow-up encompassed assessing patients’ adherence and tolerance to treatment, comfort levels while wearing the ventilator, discomfort experienced, and treatment efficacy in promptly addressing any issues encountered during the course of treatment. Participants adherent to CPAP were defined as those who received CPAP therapy for a minimum of 4 h per night on more than 70% of nights. CPAP compliance data, including device usage and hours of use per night at the prescribed pressure, were automatically recorded by the CPAP device and extracted using the appropriate software. Subsequently, after 3 months, the pain intensity of these participants was reassessed using the Short Form McGill Pain Questionnaire (SF-MPQ).

### Statistical analysis

The statistical analysis was conducted using SPSS software (version 25.0; IBM Corp, Armonk, NY, USA). Descriptive data were presented as percentages. Normally distributed continuous variables were reported as mean values with standard deviations (SD), while non-normally distributed continuous variables were described as medians with interquartile ranges (IQR). Group comparisons for continuous measurements were performed using independent t-tests, Mann–Whitney U tests, paired t-tests or Wilcoxon rank-sum tests. Categorical data were analyzed using the Chi-Square test. A multivariate logistic regression analysis was carried out to identify risk factors for pain by including significant variables from the univariate analysis. The correlations analyses of pain intensity were examined using Spearman’s correlation. Multiple linear regression was employed to assess potential independent factors predicting the most significant factor influencing pain intensity in OSA patients. *p* < 0.05 was considered significant.

### Ethical approval

This study was approved by the Ethics Committee of the Second Xiangya Hospital of Central South University (2019-215). All methods were performed in accordance with the Declaration of Helsinki. Written informed consent was obtained from all subjects.

## Results

### Demographics, sleep parameters and pain characteristics in OSA patients and healthy controls

**Figure 1:**
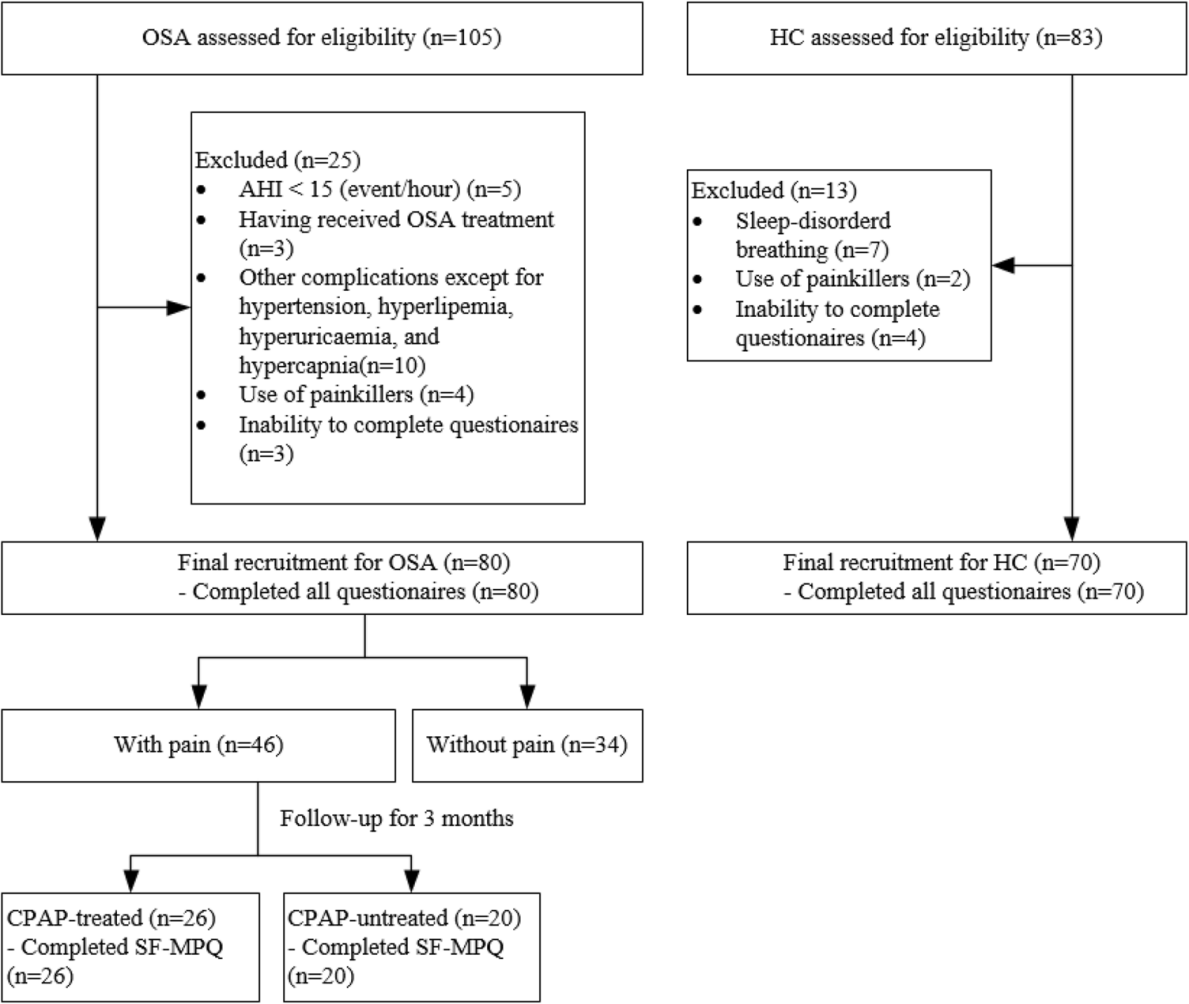
Flowchart of the participant recruitment process. OSA, obstructive sleep apnea; HC, healthy controls; AHI, apnoea hypopnea index; CPAP, continuous positive airway pressure; SF-MPQ, the Short Form McGill Pain Questionnaire.

From September 2021 to September 2022, a total of 105 potential subjects with OSA and 83 healthy controls were approached. Eventually, a total of 80 subjects with OSA and 70 healthy control subjects were recruited (Fig. 1). Among all subjects, the average age was 47 ± 12 years, 82.0% were men, and 45.3% were smokers. There were no differences in age, sex or smoking between the two groups, while BMI was higher in the OSA group (*p < *0.001). The ESS scores, AHI (apnea hypopnea index), REM-AHI (apnea hypopnea index during rapid eye movement stage), NREM-AHI (apnea hypopnea index during non-rapid eye movement stage), LSpO2 (lowest pulse oximetry), MSpO2 (mean pulse oximetry), ODI (oxygen desaturation index), N3 period, sleep efficiency, and arousal index exhibited significant differences between the OSA group and HC group (all p < 0.05) (Table 1).

**Table 1:** Demographics, sleep parameters and pain characteristics of OSA patients and healthy controls.

Variables	Total N = 150	OSA N = 80	HC N = 70	*p* value
Demographics				
Age (years)	47 ± 12	48 ± 13	46 ± 10	0.348
Gender (male) (%)	123 (82.0)	67 (83.8)	56 (80.0)	0.551
BMI (kg/m^2^)	26.3 ± 4.8	28.0 ± 4.7	24.4 ± 3.3	** < 0.001**
Smoker (%)	68 (45.3)	38 (47.5)	30 (42.9)	0.569
Sleep parameters				
ESS	4.0 (2.0–9.3)	8.0 (6.0–12)	2.0 (1.8–3.0)	** < 0.001**
AHI (events/hour)	15.5 (4.2–60.2)	45.8 (23.8–82.1)	3.9 (3.3–4.5)	** < 0.001**
REM-AHI (events/hour)	10.2 (2.7–48.6)	44.6 (16.9–60.8)	2.7 (1.8–3.4)	** < 0.001**
NREM-AHI (events/hour)	15.2 (4.2–56.0)	43.7 (19.0–78.6)	3.9 (3.3–4.5)	** < 0.001**
LSpO2 (%)	88.5 (79.5–92.0)	82.0 (71.0–88.0)	92.0 (89.8–93.3)	** < 0.001**
MSpO2 (%)	90.0 (79.5–95.3)	95.0 (93.0–97.0)	96.0 (95.0–97.0)	** < 0.001**
ODI (events/hour)	7.5 (2.5–48.6)	39 .0(16.7–75.0)	2.4 (1.7–4.3)	** < 0.001**
N3 period (%)	10.6 (4.0–16.7)	5.9 (2.0–10.2)	17.3 (13.1–20.0)	** < 0.001**
REM period (%)	17.5 ± 6.8	16.6 ± 7.4	19.6 ± 4.8	0.079
Sleep efficiency (%)	85.2 (75.9–90.1)	80.3 (68.7–89.2)	87.6 (85.6–90.6)	**0.002**
Arousal Index (events/hour)	19.2 (10.1–42.7)	30.7 (19.5–53.8)	9.8 (7.6–12.4)	** < 0.001**
Pain characteristics				
Pain (%)	65 (43.3)	46 (57.5)	19 (27.1)	** < 0.001**
Headache (%)	26 (17.3)	23 (28.8)	3 (4.3)	** < 0.001**
Pain intensity				
PRI score	3.5 (2.0–5.0)	4.0 (2.0–5.8)	2.0 (1.0–2.0)	** < 0.001**
VAS score	4.0 (2.0–6.0)	4.0 (4.0–6.0)	2.0 (2.0–4.0)	**0.011**
PPI score	0.0 (0.0–1.0)	1.0 (0.0–1.0)	0.0 (0.0–0.0)	0.117
PPI rank				
No pain (%)	33 (50.8)	21 (45.7)	12 (63.2)	0.117
Mild Discomfort (%)	26 (40.0)	19 (41.3)	7 (36.8)	
Discomfort or worse (%)	6 (9.2)	6 (13.0)	0	

Significant values are in [bold].

For comparison, χ^2^ test was used for binary variables, and Student’s t-test or Wilcoxon nonparametric test was employed for continuous variables; the italic p-values indicate statistical significance.

OSA, obstructive sleep apnea; HC, healthy controls; BMI, body mass index; ESS, Epworth sleepiness score; AHI, apnea hypopnea index; REM-AHI, apnea hypopnea index during rapid eye movement stage; NREM-AHI, apnea hypopnea index during non-rapid eye movement stage; LSpO2, lowest pulse oximetry; MSpO2, mean pulse oximetry; ODI, oxygen desaturation index; PRI, pain rank index; VAS, visual an analogue scales; PPI, present pain intensity.

**Figure 2:**
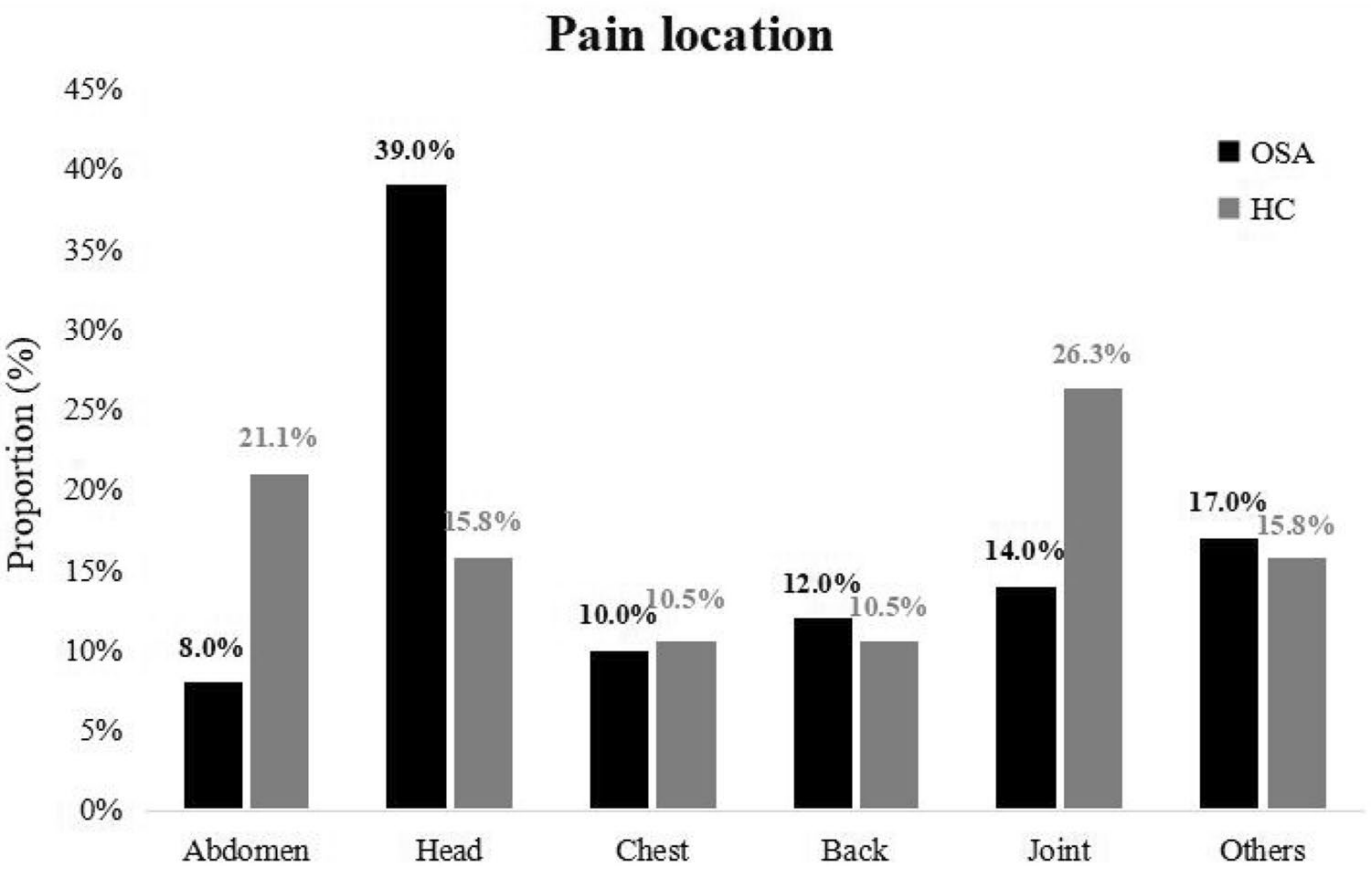
Pain location in all participants. OSA, obstructive sleep apnea; HC, healthy controls.

According to the SF-MPQ, the prevalence of pain was 46 (57.5%) in the OSA group compared to 19 (27.1%) in the healthy control group (*p* < 0.001) (Table 1). The proportion of pain locations in the OSA group and HC group showed significant differences, as shown in Fig. 2. In the HC group, pain locations were distributed more evenly. However, in subjects with OSA, the head accounted for the highest proportion of overall pain locations (39.0%), followed by joint (14.0%), back (12.0%), chest (10.0%), abdomen (8.0%), and other locations (17.0%). Additionally, the prevalence of headache was significantly higher in the OSA group with 23 cases (28.8%), as opposed to only 3 cases (4.3%) in the healthy control group (*p* < 0.001). The median pain rank index (PRI) score was 4.0 (2.0–5.8) in the OSA group versus 2.0 (1.0–2.0) in the HC group (*p* < 0.001). The median visual analogue scale (VAS) score was 4.0 (4.0–6.0) in the OSA group versus 2.0 (2.0–4.0) in the HC group (*p* = 0.011), while the present pain intensity (PPI) score and PPI rank did not show an obvious difference between the two groups (Table 1).

### Comparison between OSA patients with and without pain

In the OSA group (Table 2), the average age was 48 ± 13 years, 67 (83.8%) were male, and the average BMI was 27.9 ± 4.8 kg/m^2^, without a difference between the pain and no-pain groups. There was no obvious difference in comorbidities or sleep parameters (including ESS, AHI, REM-AHI, NREM-AHI, LSpO2, MSpO2, ODI, N3 period, REM period, sleep efficiency and arousal index) in the OSA patients with or without pain. The patients with OSA and pain had higher Hospital Anxiety and Depression Scale (HADS) scores than the patients with OSA but without pain. The median HADS anxiety score was 6.0 (4.0–7.0) in OSA patients with pain versus 4.0 (2.3–4.8) in OSA patients without pain (*p* < 0.001), and the HADS depression score was 5.0 ± 2.4 in OSA patients with pain versus 3.4 ± 2.3 in OSA patients without pain (*p* = 0.017). From the different dimensions of HRQoL measured by the SF-36, HRQoL was significantly impaired in patients with OSA and pain, which was reflected in the SF total score (106.7 (95.6–124.2) in OSA patients with pain, 129.1 (124.5–133.8) in OSA patients without pain, *p* < 0.001) and performed in physical functioning (*p* = 0.014), bodily pain (*p* < 0.001), general health (*p* < 0.001), vitality (*p* = 0.01), social functioning (*p* = 0.01), role emotional (*p* = 0.008), mental health (*p* = 0.002), and health transition (*p* = 0.015).

**Table 2:** Clinical characteristics and symptom burden in OSA patients with or without pain.

Variables	All patients N = 80	With pain N = 46	Without pain N = 34	*p* value
Demographics				
Age (years)	48 ± 13	50 ± 12	46 ± 14	0.327
Gender (male) (%)	67 (83.8)	37 (80.4)	30 (88.2)	0.35
BMI (kg/m^2^)	27.9 ± 4.8	27.5 ± 4.9	28.6 ± 4.5	0.397
Smoker (%)	38 (47.5)	19 (41.3)	19 (55.9)	0.197
Comorbidities				
Hypertention (%)	38 (47.5)	26 (56.5)	12 (35.3)	0.06
Hyperlipemia (%)	20 (25.0)	11 (23.9)	9 (26.5)	0.794
Hyperuricemia (%)	41 (51.3)	25 (54.3)	16 (47.1)	0.519
Hypercapnia (%)	19 (23.8)	10 (21.7)	9 (26.5)	0.623
Sleep parameters				
ESS	8.0 (6.0–12)	9.0 (6.5–12)	6.0 (4.3–12)	0.499
AHI (events/hour)	45.8 (23.8–82.1)	55.7 (25.1–81.8)	35.6 (18.4–83.7)	0.72
REM-AHI (events/hour)	41.4 ± 26.4	42.9 ± 22.8	38.3 ± 30.9	0.56
NREM-AHI (events/hour)	43.7 (19.0–78.6)	51.8 (18.7–78.9)	40.3 (19.5–78.3)	0.675
LSpO2 (%)	82.0 (71.0–88.0)	82.5 (67.0–86.3)	81.5 (73.0–89.5)	0.674
MSpO2 (%)	95.0 (93.0–97.0)	95 (93.3–97.0)	95.0 (93.0–96.0)	0.646
ODI (events/hour)	39.0 (16.7–75.0)	41.3 (17.3–65.0)	32.6 (12.2–75.8)	0.893
N3 period (%)	5.9 (2.0–10.2)	5.8 (1.2–11.9)	5.5 (3.2–10.1)	0.613
REM period (%)	16.5 ± 7.4	15.9 ± 7.1	17.4 ± 7.8	0.483
Sleep efficiency (%)	80.3 (68.7–89.2)	81.4 (61.1–90.6)	80.7 (72.5–88.7)	0.492
Arousal Index (events/hour)	30.7 (19.5–53.8)	34.1 (17.7–65.1)	27.3 (20.7–47.8)	0.51
HAD anxious	5.0 (4.0–7.0)	6.0 (4.0–7.0)	4.0 (2.3–4.8)	** < 0.001**
HAD depression	4.5 ± 2.5	5.0 ± 2.4	3.4 ± 2.3	**0.017**
SF-36				
Physical functioning	28.0 (26.0–30.0)	27.5 (22.8–29.8)	29.0 (28.0–30.0)	**0.014**
Role physical	7.0 (4.0–8.0)	6.5 (4.0–8.0)	7.5 (6.0–8.0)	0.146
Bodily pain	9.2 (8.1–12.0)	8.2 (6.5–9.2)	12.0 (12.0–12.0)	** < 0.001**
General health	16.4 ± 3.9	14.7 ± 3.5	18.7 ± 2.9	** < 0.001**
Vitality	18.0 (14.0–21.0)	16.0 (12.3–19.8)	19.0 (18.0–21.0)	**0.01**
Social functioning	9.0 (8.0–10)	9.0 (7.0–10)	9.5 (9.0–10)	**0.01**
Role emotional	6.0 (4.0–6.0)	5.5 (4.0–6.0)	6.0 (6.0–6.0)	**0.008**
Mental health	22.0 (19.0–26.0)	21.5 ± 3.9	24.5 ± 2.8	**0.002**
Health Transition	2.0 (2.0–3.0)	2.0 (2.0–3.0)	3.0 (2.0–3.0)	**0.015**
SF total score	123.2 (98.1–129.0)	106.7 (95.6–124.2)	129.1 (124.5–133.8)	** < 0.001**

Significant values are in [bold].

For comparison, χ^2^ test was used for binary variables, and Student’s t-test or Wilcoxon nonparametric test was employed for continuous variables; the italic p-values indicate statistical significance.

BMI, body mass index; ESS, Epworth sleepiness score; AHI, apnea hypopnea index; REM-AHI, apnea hypopnea index during rapid eye movement stage; NREM-AHI, apnea hypopnea index during non-rapid eye movement stage; LSpO2, lowest pulse oximetry; MSpO2, mean pulse oximetry; ODI, oxygen desaturation index; HADS, Hospital Anxiety and Depression Scale; SF-36, Short Form-36.

### Comparison between OSA patients with and without headache

The clinical characteristics of patients with obstructive sleep apnea (OSA) were analyzed, comparing those with and without headache. However, no significant variables, including demographics, hypercapnia, comorbidities, and sleep parameters, were found between OSA patients with and without headache (Table 3).

**Table 3:** Clinical characteristics of individuals with or without headache in patients with OSA and pain.

Variables	With headache N = 23	Without headache N = 23	*p* value
Demographics			
Age (years)	51.3 ± 12.9	47.7 ± 9.9	0.408
Gender (male) (%)	18 (78.3)	19 (82.6)	0.710
BMI (kg/m^2^)	28.0 ± 4.7	26.8 ± 5.1	0.481
Smoker (%)	10 (43.5)	9 (39.1)	0.765
Comorbidities			
Hypertention (%)	15 (65.2)	11 (47.8)	0.234
Hyperlipemia (%)	6 (26.1)	5 (21.7)	0.730
Hyperuricemia (%)	14 (60.9)	11 (47.8)	0.475
Hypercapnia (%)	6 (26.1)	4 (17.4)	0.475
Sleep parameters			
ESS	9.4 ± 3.8	8.5 ± 7.0	0.678
AHI (events/hour)	55.6 ± 35.8	54.5 ± 28.8	0.926
REM-AHI (events/hour)	46.9 ± 22.1	37.5 ± 23.5	0.291
NREM-AHI (events/hour)	54.4 ± 38.4	50.2 ± 30.0	0.754
LSpO2 (%)	58.3 (46.6–78.5)	75.3 (59.5–81.0)	0.904
MSpO2 (%)	93.0 (85.1–95.0)	93.3 (91.0–94.5)	0.572
ODI (events/hour)	45.4 ± 38.2	45.5 ± 31.2	0.995
N3 period (%)	7.0 ± 6.3	7.0 ± 6.9	0.980
REM period (%)	16.4 ± 7.3	15.3 ± 7.1	0.673
Sleep efficiency (%)	64.0 (51.1–85.4)	58.6 (37.2–73.7)	0.458
Arousal Index (events/hour)	40.1 ± 28.2	39.9 ± 26.5	0.990

For comparison, χ^2^ test was used for binary variables, and Student’s t-test or Wilcoxon nonparametric test was employed for continuous variables; the italic p-values indicate statistical significance.

BMI, body mass index; ESS, Epworth sleepiness score; AHI, apnea hypopnea index; REM-AHI, apnea hypopnea index during rapid eye movement stage; NREM-AHI, apnea hypopnea index during non-rapid eye movement stage; LSpO2, lowest pulse oximetry; MSpO2, mean pulse oximetry; ODI, oxygen desaturation index.

### Risk factors for pain in OSA patients

A multivariate logistic regression analysis was carried out to identify risk factors for pain by including significant variables from univariate analysis, which is presented in Table 4. Each demographic variables and sleep parameter were categorized into different subgroups for both univariate and multivariate analyses. For continuous variables that have no standard grouping criteria, we grouped these variables by the median of all OSA patients in the study. These variables include REM-AHI (divided line as the median (44.6 events/hour), NREM-AHI (divided line as the median (43.7 events/hour) and ODI (divided line as the median (39.0 events/hour). Other variables were grouped according to standard grouping criteria, which include age (divided line as 65 years)^18^, BMI (divided line as 28.0 kg/m^2^), AHI (divided line as 60 events/hour), LSpO2 (divided line as 80%), arousal index (divided line as 30.0 events/hour), N3 period (divided line as 12.0%), REM period (divided line as 20.0%) and sleep efficiency (divided line as 80.0%)^11^. After adjusting for ESS and LSpO2, the results of logistic regression analysis revealed that an AHI ≥ 60 events/hour was a risk factor for pain in OSA patients (OR: 7.000,* p* = 0.041).

**Table 4:** Univariate and multivariate logistic regression analysis of factors correlated with the prevalence of pain in OSA patients.

Variables	Pain (%)		Univariate			Multivariate		
	With pain N = 46	Without pain N = 34	OR	95%CI	*p* value	OR	95%CI	*p* value
Age (years)								
< 65	37 (80.4)	28 (82.4)	Ref					
≥ 65	9 (19.6)	6 (17.6)	1.135	0.362–3.562	0.828			
Gender								
Female	9 (19.6)	4 (11.8)	Ref					
Male	37 (80.4)	30 (88.2)	0.548	0.154–1.957	0.354			
Smoker								
Yes	19 (41.3)	19 (55.8)	0.556	0.227–1.361	0.199			
No	27 (58.7)	15 (44.1)	Ref					
BMI (kg/m^2^)								
< 28.0	23 (50.0)	12 (35.3)	Ref					
≥ 28.0	23 (50.0)	22 (64.7)	0.545	0.219–1.356	0.192			
ESS								
< 9.0	11 (23.9)	19 (55.9)	Ref					
≥ 9.0	35 (76.1)	15 (44.1)	4.030	1.547–10.502	**0.004**	2.879	0.574–14.438	0.199
AHI (events/hour)								
< 60	18 (39.1)	26 (76.5)	Ref					
≥ 60	28 (60.9)	8 (23.5)	5.056	1.880–13.595	**0.001**	7.000	1.078–45.437	**0.041**
REM-AHI (events/hour)								
< 44.6	14 (30.4)	16 (47.1)	Ref					
≥ 44.6	32 (69.6)	18 (52.9)	2.032	0.809–5.103	0.131			
NREM-AHI (events/hour)								
< 43.7	17 (37.0)	16 (47.1)	Ref					
≥ 43.7	29 (63.0)	18 (52.9)	1.516	0.616–3.734	0.365			
ODI (events/hour)								
< 39.0	19 (41.3)	16 (47.1)	Ref					
≥ 39.0	27 (58.7)	18 (52.9)						
LSpO2 (%)								
< 80.0%	30 (65.2)	12 (35.3)	3.437	1.358–8.703	**0.009**	0.300	0.033–2.763	0.288
≥ 80.0%	16 (34.8)	22 (64.7)	Ref					
Arousal index (events/hour)								
< 30.0	17 (37.0)	18 (52.9)						
≥ 30.0	29 (63.0)	16 (47.1)	1.263	0.517–3.086	0.608			
N3 period (%)								
< 12.0	30 (65.2)	24 (70.6)	0.781	0.301–2.031	0.612			
≥ 12.0	16 (34.8)	10 (29.4)	Ref					
REM period (%)								
< 20.0	29 (63.0)	18 (52.9)	0.659	0.268–1.264	0.365			
≥ 20.0	17 (37.0)	16 (47.1)						
Sleep efficiency (%)								
< 80.0	22 (47.8)	17 (50.0)	0.917	0.378–2.225	0.848			
≥ 80.0	24 (52.2)	17 (50.0)	Ref					

Significant values are in [bold].

BMI, body mass index; ESS, Epworth sleepiness score; AHI, apnea hypopnea index; REM-AHI, apnea hypopnea index during rapid eye movement stage; NREM-AHI, apnea hypopnea index during non-rapid eye movement stage; LSpO2, lowest pulse oximetry; MSpO2, mean pulse oximetry; ODI, oxygen desaturation index.

### Associated factors and predictors for pain intensity in OSA patients

**Figure 3:**
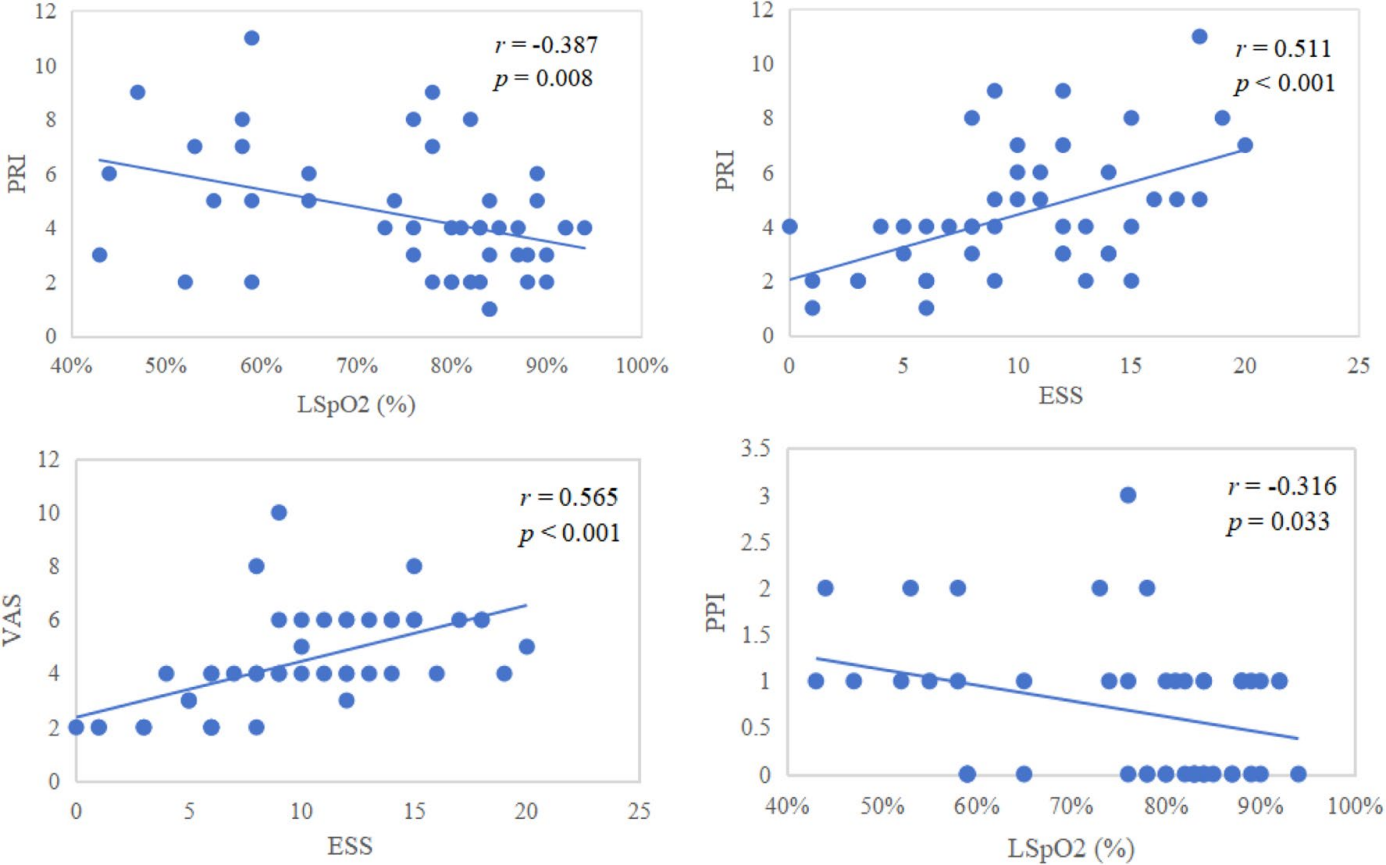
The correlation between clinical data and pain intensity in OSA patients with pain. ESS, Epworth sleepiness score; LSpO2, lowest pulse oximetry; PRI, pain rank index; VAS, visual an analogue scales; PPI, present pain intensity.

Based on the correlation analysis, in patients with OSA and pain, the PRI score exhibited a positive correlation with ESS scores (*r* = 0.511, *p* < 0.001), and a negative correlation with LSpO2 levels (*r* = − 0.387, *p* = 0.008). Furthermore, the VAS score showed a positive correlation with ESS scores (*r* = 0.565, *p* < 0.001). Additionally, the PPI score demonstrated a negative correlation with LSpO2 levels (*r* = − 0.316, *p* = 0.033) (Table 5, Fig. 3).

**Table 5:** Correlation between demographic data, sleep parameters and pain intensity in OSA patients with pain.

N = 46	PRI score		VAS score		PPI score	
	*r*	*p*	*r*	*p*	*r*	*p*
Age	0.339	0.058	0.273	0.13	0.225	0.215
BMI	0.123	0.502	0.171	0.348	0.109	0.551
ESS	0.511	**< 0.001**	0.565	**< 0.001**	0.095	0.605
AHI (events/hour)	0.171	0.348	0.257	0.155	0.107	0.561
REM-AHI (events/hour)	0.052	0.778	0.12	0.514	0.103	0.577
NREM-AHI (events/hour)	− 0.06	0.743	0.022	0.905	0.024	0.896
LSpO2 (%)	− 0.387	**0.008**	− 0.328	0.067	− 0.316	**0.033**
MSpO2 (%)	0.045	0.816	0.025	0.898	− 0.158	0.413
ODI (events/hour)	0.021	0.909	0.046	0.802	0.049	0.789
N3 period (%)	-0.173	0.343	-0.199	0.274	− 0.097	0.596
REM period (%)	0.13	0.478	0.181	0.32	0.188	0.302
Sleep efficiency (%)	− 0.184	0.315	− 0.019	0.916	− 0.005	0.98
Arousal Index (events/hour)	− 0.054	0.769	0.039	0.831	− 0.003	0.985

Significant values are in [bold].

BMI, body mass index; ESS, Epworth sleepiness score; AHI, apnea hypopnea index; REM-AHI, apnea hypopnea index during rapid eye movement stage; NREM-AHI, apnea hypopnea index during non-rapid eye movement stage; LSpO2, lowest pulse oximetry; MSpO2, mean pulse oximetry; ODI, oxygen desaturation index; PRI, pain rank index; VAS, visual an analogue scales; PPI, present pain intensity.

As is shown in Table 6, The pain intensity in OSA was predicted using a multiple linear regression model, incorporating age, sex, BMI, smoker, ESS score, and LSpO2 as independent variables. Age and ESS positively predicted the PRI score (*β* = 0.512, *p* = 0.007), while LSpO2 negatively predicted the PRI score in OSA patients (*β* = − 0.412, *p* = 0.011). Regarding the VAS score, ESS positively predicted the VAS score (*β* = 0.508, *p* = 0.007). Therefore, age, ESS, and LSpO2 may be the predictors of the pain intensity in OSA.

**Table 6:** Multiple linear regression analysis for the prediction of pain intensity in patients with OSA and pain.

N = 46	PRI score			VAS score			PPI score		
	*β*	*t*	*p*	*β*	*t*	*p*	*β*	*t*	*p*
Age (year)	0.336	2.29	**0.031**	0.178	1.215	0.236	0.171	0.938	0.357
Gender (male)	0.087	0.551	0.586	− 0.073	− 0.467	0.645	− 0.259	− 1.32	0.199
BMI (kg/m^2^)	− 0.179	− 1.139	0.265	− 0.134	− 0.859	0.399	0.065	0.949	0.784
Smoker	− 0.199	− 1.155	0.259	0.249	1.453	0.159	0.301	0.648	1.542
ESS	0.512	2.937	**0.007**	0.508	2.929	**0.007**	− 0.101	− 0.465	0.646
LSpO2 (%)	− 0.412	− 2.757	**0.011**	− 0.182	− 1.221	0.233	− 0.377	− 2.034	0.053

Significant values are in [bold].

BMI, body mass index; ESS, Epworth sleepiness score; LSpO2, lowest pulse oximetry.

### Pain intensity in CPAP-treated group and CPAP-untreated group

All OSA patients experiencing pain were followed up after three months, and the pain intensity in these patients was reassessed. Out of the total, 26 (56.5%) patients demonstrated adherence to CPAP treatment and were categorized as the CPAP-treated group, while 20 (43.5%) patients exhibited non-compliance with CPAP treatment and were classified as the CPAP-untreated group. Table 7 presents the impact of CPAP treatment on pain intensity in OSA patients with pain, along with a secondary analysis for both the CPAP-treated and CPAP-untreated groups. We observed a significant improvement in both PRI score and VAS score (all *p* < 0.001), which remained statistically significant only among adherent patients receiving CPAP therapy.

**Table 7:** Evaluation of pain intensity before and after CPAP treatment in patients with OSA and pain.

Variables	All patients (N = 46)			CPAP treated (N = 26)			CPAP untreated (N = 20)		
	Baseline	After 3 months	p*	Baseline	After 3 months	*p*^’^	Baseline	After 3 months	*p*^#^
PRI score	4.4 ± 2.3	3.3 ± 2.0	** < 0.001**	4.7 ± 2.3	2.9 ± 2.1	** < 0.001**	4.0 (2.0–5.0)	4.0 (2.3–5.0)	0.506
VAS score	4.0 (4.0–6.0)	4.0 (2.5–4.0)	** < 0.001**	6.0 (4.0–6.0)	4.0 (2.0–4.0)	** < 0.001**	4.0 (4.0–5.8)	4.0 (4.0–4.0)	0.225
PPI score	1.0 (0.0–1.0)	0.0 (0.0–1.0)	0.107	0.5 (0.0–1.0)	0.0 (0.0–1.0)	0.129	1.0 (0.0–1.0)	0.5 (0.0–1.0)	0.541

Significant values are in [bold].

CPAP, continuous positive airway pressure; PRI, pain rank index; VAS, visual an analogue scales; PPI, present pain intensity.

*Comparison between variables at baseline and after 3 months of CPAP treatment in all patients; ’ = comparison between variables at baseline and after 3 months of CPAP treatment in CPAP-treated patients; # = comparison between variables at baseline and after 3 months of CPAP treatment in CPAP-untreated patients; data were compared between two groups using paired t-test or Wilcoxon rank-sum test.

## Discussion

Patients with obstructive sleep apnea (OSA) frequently experience pain-related issues. However, limited research has been conducted in this area. Recent studies have demonstrated that OSA can contribute to the development and exacerbation of various unpleasant conditions, such as headaches, temporomandibular disorders, and fibromyalgia^4^. The comprehensive assessment of pain issues in patients with OSA and healthy controls is conducted for the first time in this study.

In a recent research, the prevalence of obstructive sleep apnea (OSA) was found to be 13.8% in a retrospective analysis conducted among patients attending a pain management clinic^19^. The findings of our study demonstrate that pain manifested as a prevalent impairment among individuals with OSA, surpassing its occurrence in healthy controls. According to multivariate logistic regression analysis, we found that very severe OSA had an increased risk for pain problems. Very severe OSA is defined as an Apnea–Hypopnea Index (AHI) ≥ 60 events per hour, which typically exhibits more pronounced nocturnal hypoxemia and symptom burden. According to a research study, individuals with severe OSA reported significantly higher rates of drowsy driving and accidents caused by falling asleep^20^. Therefore, clinicians should prioritize the management of pain in individuals with very severe OSA.

According to earlier research, patients with OSA had considerably more morning headache than patients without OSA^21^, and treating OSA resulted in clinically significant improvements in over 30% of patients with undifferentiated headache^22^. In our study, the head was found to be the most prevalent location for pain in subjects with OSA, accounting for 39% of overall pain locations. Additionally, a significant proportion (28.8%) of patients diagnosed with OSA reported experiencing headaches, suggesting a high prevalence of headache among individuals with OSA. However, upon comparing the clinical characteristics of OSA patients with and without headache, we did not identify any specific factors associated with headache in OSA patients. As reported in the literature, the impact of OSA severity on the prevalence of sleep apnea headache is minimal. The underlying mechanisms behind sleep apnea headache remain elusive, as there are no significant differences in average oxygen desaturation and lowest oxygen saturation between OSA patients with or without headache^23^.

The term “health-related quality of life” (HRQoL) refers to how one's health, encompassing physical, psychological, and social functions, affects their level of life satisfaction and pleasure^10^. The findings of a comparative mixed-methods study indicate that individuals experiencing pain exhibit lower health-related quality of life (HRQoL) compared to both the general population and patients with other chronic conditions, owing to the multifaceted adverse effects associated with pain^24^. We observed that patients with obstructive sleep apnea (OSA) and pain exhibited significantly poorer health-related quality of life (HRQoL) and psychological well-being compared to those without pain. The SF-36 questionnaire revealed substantial impairments in mental health, bodily pain, vitality, and role emotional domains among these patients, which were further supported by the results obtained from the SF-MPQ and HADS assessments. These findings underscore the detrimental impact of pain on HRQoL in individuals with OSA, emphasizing the need for clinicians to prioritize this comorbidity and consider screening for OSA in patients experiencing pain.

It is unclear exactly how OSA and pain are related. According to a theory^6^ put forth, OSA’s overall impact on pain is a culmination of the effects of sleep deprivation, inflammatory mediators, and hypoxia markers. These effects increase pain sensitivity, that contributes to neuronal pain sensitivity, and increase spontaneous pain^25,26^. The mechanisms in OSA result in sleep fragmentation and nocturnal hypoxemia. These two changes in OSA patients may be involved in the development of a number of OSA comorbidities (e.g., excessive daytime sleepiness) and molecular changes (e.g., pain sensitization)^27^. We further found that the associated factors for pain intensity in OSA included age, ESS ≥ 9.0 and lowest pulse oximetry (LSpO2) < 80.0%. Many patients with OSA experience excessive daytime sleepiness (EDS), which can negatively affect daily functioning, cognition, mood, and other aspects of well-being. The Epworth Sleepiness Scale (ESS) is an eight-item self-report measure used to assess trait excessive daytime sleepiness (EDS), where patients rate their propensity for dozing off or falling asleep in various scenarios such as reading, watching television, or riding in a car; an ESS score of ≥ 9.0 indicates the presence of excessive daytime sleepiness^28^. Hypoxemia in OSA has been proven to have a significant impact on increased expression of proinflammatory cytokines influencing the hyperalgesic priming of nociceptors. Moreover, hypoxia markers by themselves are hypothesized to modulate intracellular signal transduction in neurons and have an impact on nociceptive sensitization^6^. We found that the pain intensity of OSA patients was also related to worse pulse oximetry and severe nocturnal hypoxemia may be one of the predictors of pain intensity in OSA. These results indicated that the degree of daytime sleepiness and nocturnal hypoxia, may play an important role in the development of pain in OSA.

The use of continuous positive airway pressure (CPAP) is an effective treatment for OSA because CPAP normalizes sleep architecture, reduces subjective excessive daytime sleepiness and reverses other OSA symptoms^29^. CPAP treatment prevents hypoxia and oxygenation injury and inhibits the cascade of deleterious OSA-induced consequences^30^. Patients with severe OSA and metabolic syndrome who exhibit good compliance with CPAP treatment may exhibit improved insulin sensitivity and reduced systemic inflammation, and global cardiovascular disease risk^31^. Previous studies have demonstrated that severe OSA patients are hyperalgesic, their pain sensitivity improves with CPAP treatment, and the improvement disappears immediately with discontinuation of CPAP therapy^32^. In patients with moderate-severe OSA at high risk of cardiovascular events and without severe sleepiness, CPAP improved daytime sleepiness and multiple domains of HRQoL over 6–12 months of follow-up, with the largest improvement observed in bodily pain^33^. The results of this study show that in patients with moderate-to-severe OSA and pain, good compliance with CPAP treatment can improve pain intensity. Therefore, OSA patients should be evaluated for pain and receive timely intervention, and there should be a focus on education for OSA patients with pain to increase compliance with CPAP treatment.

To the best of our knowledge, this study represents the first attempt to investigate the prevalence, distribution, intensity, nature, and associated factors of pain and its severity in patients with OSA. However, it is important to acknowledge certain limitations in our study. Firstly, the study was conducted at a single center with a limited sample size, predominantly consisting of male patients, which may limit the generalizability to the overall OSA population. Secondly, it is important to consider that comorbidities such as hypertension, hyperuricemia, and hypercapnia, which have the potential to influence pain assessment in OSA patients, were not specifically excluded from this study. Lastly, it is worth mentioning that participants' verbal comprehension and subjective experiences might have influenced their responses on the questionnaire ratings, particularly regarding the visual analog scale (VAS) score.

## Conclusion

Pain was prevalent among patients with moderate-to-severe OSA and was associated with depression, anxiety, and a lower HRQoL. Patients with very severe OSA had an increased risk for pain problems. The intensity of pain in OSA can be predicted by age, ESS ≥ 9.0, and LSpO2 < 80.0%, and it can be alleviated through CPAP treatment.

## Data Availability

The datasets analysed during the current study are available from the corresponding author on reasonable request.

## Abbreviations

OSA: Obstructive sleep apnea
HC: Healthy controls
BMI: Body mass index
SF-MPQ: The Short Form McGill Pain Questionnaire
BPI: The Brief Pain Inventory
HADS: Hospital Anxiety and Depression Scale
SF-36: Short Form-36
HRQoL: Health-related quality of life
PRI: Pain rank index
VAS: Visual an analogue scales
PPI: Present pain intensity
ESS: Epworth sleepiness score
AHI: Apnea hypopnea index
REM-AHI: Apnea hypopnea index during rapid eye movement stage
NREM-AHI: Apnea hypopnea index during non-rapid eye movement stage
ODI: Oxygen desaturation index
LSpO2: Lowest pulse oximetry
MSpO2: Mean pulse oximetry
CPAP: Continuous positive airway pressure
